# Modeling optimal cervical cancer prevention strategies in Nigeria

**DOI:** 10.1186/1471-2407-14-365

**Published:** 2014-05-24

**Authors:** Nadia Demarteau, Imran O Morhason-Bello, Babatunde Akinwunmi, Isaac F Adewole

**Affiliations:** 1 Health Economics, Global Vaccines Development, GlaxoSmithKline Vaccines, Avenue Fleming 20 B-1300, Wavre, Belgium; 2 Department of Obstetrics & Gynaecology, College of Medicine, University of Ibadan/University College Hospital, Ibadan, Oyo State, Nigeria

**Keywords:** CC, Human papillomavirus vaccination, Optimization model, Africa, Nigeria

## Abstract

**Background:**

This study aims to assess the most efficient combinations of vaccination and screening coverage for the prevention of cervical cancer (CC) at different levels of expenditure in Nigeria.

**Methods:**

An optimization procedure, using a linear programming approach and requiring the use of two models (an evaluation and an optimization model), was developed. The evaluation model, a Markov model, estimated the annual number of CC cases at steady state in a population of 100,000 women for four alternative strategies: screening only; vaccination only; screening and vaccination; and no prevention. The results of the Markov model for each scenario were used as inputs to the optimization model determining the optimal proportion of the population to receive screening and/or vaccination under different scenarios. The scenarios varied by available budget, maximum screening and vaccination coverage, and overall reachable population.

**Results:**

In the base-case optimization model analyses, with a coverage constraint of 20% for one lifetime screening, 95% for vaccination and a budget constraint of $1 per woman per year to minimize CC incidence, the optimal mix of prevention strategies would result in a reduction of CC incidence of 31% (3-dose vaccination available) or 46% (2-dose vaccination available) compared with CC incidence pre-vaccination. With a 3-dose vaccination schedule, the optimal combination of the different strategies across the population would be 20% screening alone, 39% vaccination alone and 41% with no prevention, while with a 2-dose vaccination schedule the optimal combination would be 71% vaccination alone, and 29% with no prevention. Sensitivity analyses indicated that the results are sensitive to the constraints included in the optimization model as well as the cervical intraepithelial neoplasia (CIN) and CC treatment cost.

**Conclusions:**

The results of the optimization model indicate that, in Nigeria, the most efficient allocation of a limited budget would be to invest in both vaccination and screening with a 3-dose vaccination schedule, and in vaccination alone before implementing a screening program with a 2-dose vaccination schedule.

## Background

The incidence of cervical cancer (CC) in the Sub-Saharan Africa region, where Nigeria is located, is among the highest in the world. The CC incidence per 100,000 in Sub-Saharan Africa is 19.1 [1], whereas the world average rate is 15.2 per 100,000. CC death rates are also high in Sub-Saharan Africa, with rates per 100,000 of 12.8, compared with the world average of 7.8 per 100,000. In Sub-Saharan countries, CC is either the most common cancer in women or the second most common cancer (after breast cancer) in women [1].

In many developed countries, where national routine screening programs using the Papanicolaou (Pap) smear have been implemented, the CC incidence and mortality have been significantly reduced [2-5]. Early detection and treatment of cervical precancerous lesions is associated with high cure rates, whereas failure to detect precancerous lesion increase the risk to CC development and hence the risk of premature death. In many Sub-Saharan African countries, there are currently no programs for mass CC screening and even when such programs are set up in family planning clinics they are perceived as cumbersome and underutilized [6,7].

Vaccination provides an alternative or a supplementary intervention for CC prevention. Infection with human papillomavirus (HPV) has been shown to be a necessary condition for the development of CC [8-10]. Eight HPV genotypes (HPV 16, 18, 45, 31, 33, 52, 58, and 35) account for more than 90% of CC cases, with HPV 16 and 18 accounting for about 70% of cases worldwide [11,12]. Two HPV vaccines are currently available, an AS04-adjuvanted HPV-16/18 vaccine and a HPV-6/11/16/18 L1 virus-like particle vaccine that covers two non-oncogenic HPV types (HPV 6 and 11), as well as the oncogenic types HPV 16 and 18. Both vaccines have an efficacy of approximately 98% against the HPV 16 and 18 genotypes, but have different levels of cross-protection against other oncogenic HPV types [13-15]. The currently approved schedule for the available vaccines requires three doses over a 6-month time period for optimum efficacy and is generally given before the onset of sexual activity [16,17]. However, recent studies have indicated that two doses of vaccine may be sufficient and the 2-dose schedule was consequently registered in different countries worldwide including Nigeria [18,19]. The full long term duration of protection has not been fully determined as yet, but sustained immunogenicity and efficacy have been shown for up to 9.4 years for the HPV 16/18 AS04 adjuvanted vaccine [20]. Also, a conservative estimate from a modeling exercise estimated that the antibody levels would remain well above levels induced by natural infection for at least 50 years [21]. Even though the correlate of protection is unknown, neutralizing antibodies are considered to be the primary mechanism of vaccine-induced protection, hence these results potentially indicate long term protection with the vaccine.

Numerous studies have investigated the cost-effectiveness of HPV vaccination or CC screening in many countries in Europe, Africa, and Latin America, and most have concluded that both methods of prevention are cost-effective.

Standard cost-effectiveness or budget-impact analyses are however not the best methods to determine which mix of prevention strategies provides the most efficient use of limited resources. Standard cost-effectiveness analyses do not typically take into account affordability constraints when estimating the cost-effectiveness of different combinations of prevention strategies, and are also limited in their ability to examine the comparative efficiency of many different combinations of prevention interventions. Because neither vaccination nor screening alone can provide 100% protection against CC, an optimal prevention strategy might include a combination of both. Budget-impact analyses typically estimate only affordability and do not link budget impact to health outcomes.

An alternative approach to economic assessment is optimization modeling applied previously in many different areas such as transport, agriculture, industry, and banking [22], and more recently in the health care sector [23-28]. This approach uses mathematical programming techniques to select the combination of alternative interventions that achieves the best clinical outcome while meeting pre-selected constraints on the available budget and on the feasibility of different coverage levels for the alternative interventions.

Optimization modeling provides valuable additional information compared with either cost-effectiveness or budget-impact analysis, since it explicitly evaluates multiple available options to select the combination that fulfills all the constraints introduced in the model while obtaining the most efficient result: lowest cost for the best outcome [22-28]. This is especially suitable for assessing public health interventions, where large but limited budgets must be allocated among different intervention options to allow a specific health goal to be reached. Compared with cost-effectiveness analysis for decision-making, optimization modeling does not require a pre-specified cost-effectiveness threshold, which is associated with much debate in the literature.

The goal of this analysis was to provide information for Nigeria, as an example of a Sub-Saharan African country currently investigating a solution to improve CC prevention, about the most efficient combinations of prevention and screening coverage at different levels of expenditure. Nigeria has a population of about 170 million and is also the most populous country in Africa with a high CC burden [29]. Moreover, women in Nigeria typically present at an advanced CC stage; at least 80% present with stage III disease and 10% with stage IV disease based on the Classification of Malignant Tumours (TNM), accounting for the observed high mortality rates [7].

We used an optimization model to identify the combinations of vaccination and screening coverage that would provide the greatest estimated reduction in the annual CC incidence for different levels of expenditure per woman in Nigeria. This information can be used by policy-makers in Nigeria and other countries in Africa with similar CC incidence and mortality rates when designing strategies to reduce the CC burden in their country.

## Methods

The optimization model used in this study to identify the optimal combination of CC prevention strategies in Nigeria has been applied previously to the United Kingdom (UK) and Brazil to run similar analyses [30]. This evaluation estimates the optimal mix of CC prevention strategies to be implemented annually under specific constrains at steady state to minimize CC incidence. The steady state, in this evaluation, refers to a year over which, following the implementation of the prevention strategy in the entire population, all the benefits as well as the associated costs are captured across the entire population.

The optimization procedure requires the use of two models. The first model embedded within the optimization procedure was a Markov cohort model: the “evaluation” model. It was used to generate estimates of the annual incident CC cases at steady state in a population of 100,000 women for each of four alternative strategies considered in the evaluation: screening only; vaccination only; screening plus vaccination; and no prevention. The number of incident CC cases was chosen as the primary outcome measure because CC prevention represents the ultimate goal of screening or vaccination. The results of the Markov model for each scenario were used as inputs to the optimization model. The optimization model was then used to determine the optimal mix of interventions for maximizing the reduction in CC incidence under different scenarios. Those scenarios varied by available budget and by constraints on the maximum screening and vaccination coverage to be reached and the overall reachable population. Alternative scenarios were considered by varying the screening and vaccination coverage constraints to model different feasible intervention uptakes within a Sub-Saharan African country such as Nigeria. The purpose of testing different budget scenarios was to reflect the reality of limited health care funding and to demonstrate the incremental reduction in CC cases that would be possible with additional spending. This evaluation is intended to inform decision-makers about the health and economic impact of different prevention strategies as well as the optimal potential program.

### Evaluation model

**Figure 1:**
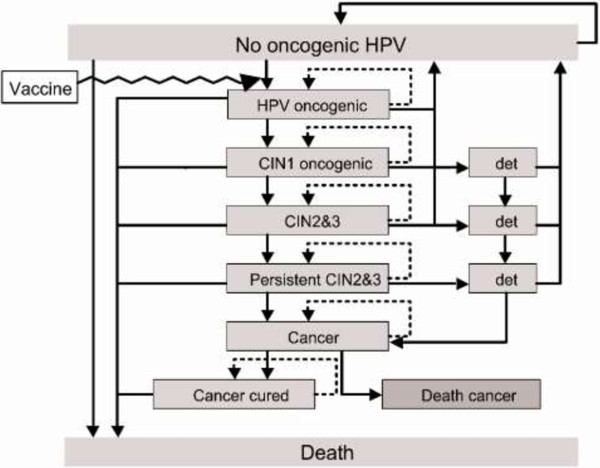
**Markov model flow diagram.** Source: [32]. HPV: Human papillomavirus; CIN: Cervical intraepithelial neoplasia; Det: Lesion detected by the screening.

A previously developed Markov cohort model built in Microsoft *Excel* was adapted to the Nigerian setting and was used to estimate the clinical and cost outcomes associated with each specified prevention strategy analyzed separately among a female population [31-33]. Screening was assumed to be cytology-based. Eight strategies were analyzed using the Markov model: one lifetime screening at age 35 years; two lifetime screenings at ages 35 and 40 years; three lifetime screenings at ages 35, 40, and 45 years; vaccination of girls at age 12 years; vaccination and one lifetime screening; vaccination and two lifetime screenings; vaccination and three lifetime screenings; and no prevention. The screening strategies (one, two or three lifetime screenings) were selected to reflect the screening programs that could be implemented in Nigeria and other Sub-Saharan African countries.The Markov model consisted of 12 health states, reflecting the natural history of the disease and the effect of screening and vaccination: no infection with an oncogenic HPV virus; infection with an oncogenic HPV virus without precancerous or cancerous lesion; cervical intraepithelial neoplasia (CIN) grade 1; CIN grade 2 or 3; persistent CIN grade 2 or 3; CC; diagnosed CIN grade 1 through screening; diagnosed CIN grade 2 or 3 through screening; diagnosed persistent CIN grade 2 or 3 through screening; CC cured; death from CC; and death from other causes (see Figure 1).

The natural history transition rates between the different model health states were assumed to be the same as those used in the original Markov model [32] and were based on published natural history studies (Table 1). The other input values were adapted to reflect the epidemiology and costs of CC in Nigeria whenever available, or in the continent of Africa if country-specific data were not available [30-33]. In particular, the incidence of HPV infection was taken from a study of the prevalence of HPV infection in Nigerian women, converted to incidence data based on natural mortality in Nigeria, HPV regression and HPV progression rate [34].

**Table 1:** Markov model inputs

**Variable**	**Input value**	**Data source**
***Vaccination***		
Vaccine coverage	50% to 95%	Assumption
Vaccine efficacy 16-18	98%	[35,36]
Proportion of HPV 16/18 related CIN1, CIN2/3 and CC	22%, 41%, 50%	[37]
Vaccine efficacy 10 other HPV oncogenic types	68.3%	[36,38]
Proportion of 10 other HPV type related CIN1, CIN2/3 and CC	24%, 35% 32%	[37]
Age at vaccination	12 years	Assumption
Vaccine waning	None	Assumption
***Screening***		
Once—age	35 years	Assumption
Twice—ages	35, 40 years	Assumption
Three times—ages	35, 40, 45 years	Assumption
Percentage screened	20% to 40%	Assumption
Cytology sensitivity CIN1	70%	[39]
Cytology sensitivity CIN2/3	80%	[39]
Compliance with CIN1 treatment	100%	[39]
Compliance with CIN2/3 treatment	100%	[39]
Efficacy of CIN treatment	95%	[39]
Cost (2011 US dollars)*		
Vaccine cost per dose	2365 NGN ($15)	PAHO price
Cytology test	3010 NGN ($16)	[40]
Colposcopy + biopsy	7225 NGN ($46)	[40]
Annual CIN1 treatment *(average resources used per patient: 1.7 consultations, 3.2 procedures, 1.1 medications, 0.5 hospitalizations)*	40,672 NGN ($258)	[40]
Annual CIN2/3 treatment *(average resources used per patient: 1.8 consultations, 4.1 procedures, 2.1 medications, 0.9 hospitalizations)*	68,200 NGN ($432)	[40]
Lifetime CC treatment cost *(average resources used per patient: 2.4 consultations, 7.1 procedures, 6.1 medications, 1.1 hospitalizations)*	227,026 NGN ($1440)	[40]
***Transition probabilities***		
Age-specific mortality rate	0.01 to 1	[41]
Uninfected to HPV	0.0 to 0.14	[34]
HPV to CIN1	0.05	[42]
CIN1 to CIN 2/3	0.09	[43-45]
CIN2/3 to persistent CIN2/3	0.11	[43,44]
HPV clearance to uninfected	0.29 to 0.55	[42,44-46]^**^
CIN1 clearance	0.45	[43-45,47,48]
CIN 2/3 clearance	0.23	[43-45,47,48]
Persistent CIN2/3 to cancer	0.0 to 0.10	Estimated from calibration to CC
Cancer to cancer cured	0.084	Estimated from 5-year survival of 34.4% Nigeria ( [1])

CIN1 = Cervical intraepithelial neoplasia, grade 1; CIN2/3 = Cervical intraepithelial neoplasia, grades 2 and 3; HPV = Human papillomavirus; NGN = Nigerian naira.

*Exchange rate used 1 NGN = $0.0063.

**Age-specific HPV clearances were used as reported in the literature.

The validity of the model was assessed by comparing the estimated age-dependent CC incidence without any prevention strategy to the CC incidence reported in GLOBOCAN [1]. Calibration to the reported CC incidence was done by adjusting the persistent CIN2/3 to CC transition probability.

Health care services used for treating CIN grade 1, CIN grades 2 and 3, and CC were taken from a retrospective chart review performed at the university college hospital at Ibadan where patients’ charts are archived. The chart review collected the medical resources used (outpatient health care professional visits, outpatient diagnostic procedures, outpatient treatment procedures, medications, and hospitalizations) to treat a patient with CIN1, CIN2/3 or CC. This study received approval from the University of Ibadan/University College Hospital Ethics Committee. For precancerous lesions, resources used over a 1-year period from the date of diagnosis were collected; for CC, lifetime resources were collected (from diagnosis until either death or cure). For each condition, 10 patients with the required information at the time of data collection (2010) were identified (using consecutive medical records) and the medical resources used were extracted from their hospital medical records. The associated costs were estimated after assigning unit costs from the hospital record to each of the medical resources used. The costs were adjusted to 2012 values based on the Nigerian consumer price index for the health care sector [40]. The average costs from all patients per condition were used as input to the model (Table 1). Health care services for screening were based on expert opinion, and unit costs were estimated based on the average unit costs for each procedure reported in the hospital records from the university college hospital at Ibadan. The cost of the vaccine program was assumed to be $15 per dose (based on the Pan American Health Organization (PAHO) price).

Vaccine efficacy was estimated as the weighted average vaccine efficacy of 98% for HPV types 16 and 18 and 68.4% for the 10 most frequent non-vaccine HPV types (HPV-31/33/35/39/45/51/52/56/58/59) related to CC, based on the clinical trial results of the AS04-adjuvanted HPV-16/18 vaccine, with weights reflecting the relative frequency of the different HPV types in Nigerian women (Table 1). Matching efficacy was assumed for both the 3-dose and the 2-dose vaccination schedule.

For each prevention strategy, the Markov model estimated the lifetime costs for prevention and treatment of CIN and CC and the lifetime incident CC cases for a cohort of women. The lifetime outcomes from the Markov model were divided by the total life-years lived by the cohort and multiplied by 100,000 to provide an estimate of 1-year values at steady state for a population of 100,000 women, assuming that the age distribution for the population remained constant over time. These outcomes were then used as inputs to the optimization model. Because the estimated Markov model results were used to estimate the steady-state, cross-sectional, 1-year values for the whole population of interest, no discount rate was applied.

### Optimization model

We used Solver (Frontline), an *Excel* add-in to solve the optimization model. In the base case, we considered only a screening frequency of once in a lifetime at age 35 years. As a result, only four prevention strategies were included: no prevention; one lifetime screening; vaccination alone; and vaccination plus one lifetime screening. The optimization model was used to estimate the proportion of the population receiving each of the CC prevention strategies that minimized the expected CC incidence, considering a fixed budget and pre-specified constraints on screening coverage, vaccine coverage, and overall reachable population. The four different CC prevention strategies were mutually exclusive. In the base-case analyses, the optimization model distributed the population between the four predefined prevention strategies in the objective function under several constraints:

Objective function:

$$\text{Minimize}\;\overset{4}{\underset{i=1}{\sum \;}}\;{\text{CC}}_{i}\;\cdot x_{i}$$

Subject to the following constraints:

$$\text{Budget constraint}:0\leq x_{i}\leq 1\;\overset{4}{\underset{i=1}{\sum }}b_{i}\cdot x_{i}\leq B$$

$$\begin{array}{l} \text{Limit}\;\text{percentages}\;\text{receiving}\;\text{strategies}\;\text{to}\;\text{be}\\ \text{between}\;0\%\;\text{and}\;100\;\%:0\leq x_{i}\leq 1\;\text{for}\;i\;=1,...,4 \end{array}$$

$$\text{Screening}\;\text{coverage}\;\text{upper}\;\text{bound}:\sum_{s=1}^{2}\;x_{s}\leq \text{Coverag}e_{1}$$

$$\text{Vaccine}\;\text{coverage}\;\text{upper}\;\text{bound}:\overset{2}{\underset{s=1}{\sum }}x_{v}\leq \text{Coverag}e_{2}$$

$$\text{Distribution}\;\text{over}\;\text{the}\;\text{entire}\;\text{population}:\sum_{i=1}^{4}\;x_{i}=1$$

$$\begin{array}{l} \text{Overall}\;\text{reachable}\;\text{population}:\\ X_{\text{nprev}}=\min\left(\left[1‒{\text{Coverage}}_{1}\right],\left[1‒{\text{Coverage}}_{2}\right]\right) \end{array}$$

With *x*_*i*_ ∈ ℝ

■ *x*_*i*_ is a continuous decision variable representing the proportion of the population in strategy *i*; these four values for *x*_*i*_ are the optimization variables *i* (*i* = 1, …, 4) that denote the four different predefined prevention strategies: no prevention, screening once, vaccination alone, and vaccination plus screening once.

■ *s* denotes a subset of the *i* strategies including screening (2 strategies out of 4, 1 with screening alone, 1 with both vaccination and screening).

■ *v* denotes a subset of the *i* strategies including vaccination (2 strategies out of 4, 1 with vaccination alone and 1 with both vaccination and screening).

■ *x*_*nprev*_ represents, among the *x*_*i*_, the proportion of the population receiving no preventive measure.

■ *Coverage*_*1*_ and *Coverage*_*2*_ denote the upper bounds for coverage for screening and vaccination, respectively; these are selected to represent either readily achievable or ideal values.

■ *CC*_*i*_ represents the number of CC cases at steady state per 100,000 women receiving prevention strategy *i* as estimated by the evaluation model.

■ *b*_*i*_ is the cost for 100,000 women at steady state receiving strategy *i* as estimated by the evaluation model.

■ *B* is the overall CC-related (prevention and treatment) budget across the population.

### Base case analyses

The pre-vaccination budget was estimated assuming that there is no national screening or vaccination program in Nigeria, the associated expenditure per woman per year being the cost of treatment for those with CC, corresponding to $0.25 per woman per year across the entire female population. In the base-case analyses, the constraint on annual expenditure per woman was increased gradually from $0.25 to $2.0, and the annual incident number of CC cases was estimated for each level of annual expenditure when the optimal combination of prevention strategies is implemented. For the two vaccination schedules 3 and 2 doses were considered. Three scenarios were estimated for the full range of budget constraints: (1) maximum screening coverage of 20% and maximum vaccination coverage of 95%; (2) maximum screening coverage of 40% and maximum vaccination coverage of 95%; and (3) maximum screening coverage of 20% and maximum vaccination coverage of 50%. These ranges were selected based on expected or targeted ranges for screening and vaccination coverage within the Nigerian setting. An additional constraint required a minimum number of people to receive no prevention. This constraint equals the lower of one minus the upper-bound coverage constraint for either screening or vaccination and hence is directly linked to the screening and vaccination coverage constraint.

### Sensitivity analyses

Univariate sensitivity analyses were conducted to estimate the effects on the incident number of CC cases for two different budget constraints, set at $1 and $2 per woman per year, when changing the costs associated with screening, vaccination, and treatment of CIN and CC. Ranges of plus or minus 20% were used for the screening costs, and ranges of plus or minus one standard deviation from the means were used for the costs of treatment of CIN and CC, using the data from the retrospective chart review. In addition, the impact on CC cases of adding strategies with more frequent screening, two lifetime screenings (at ages 35 and 40 years) or three lifetime screenings (at ages 35, 40 and 45 years), was tested. The sensitivity analysis on treatment costs accounted for setting with higher or lower costs than the one estimated in the retrospective cost evaluation that may not be representative of all settings in Nigeria. An additional scenario included the possibility of implementing an HPV test screening instead of the Pap test. In this scenario the cost of the screening test was set to 50% of the Pap test costs and CIN sensitivity was increased by an absolute value of 10%. Finally, the duration of vaccine efficacy was reduced from lifetime to 25 years, and also the vaccine efficacy was reduced by an absolute value of 20%.

## Results

### The evaluation model

Table 2 presents the outcomes, total cost, and annual incident number of CC cases for the prevention strategies used to generate inputs for the base case and alternative optimization model. These results indicate that screening all women once in a lifetime ($75,418 per 100,000 women) or providing no prevention ($26,201 per 100,000 women) is less expensive than vaccinating all women ($191,415 and $130,603 per 100,000 women for a 3- and 2-dose vaccine, respectively). One lifetime screening is less effective than vaccination at reducing incident CC cases (12.15 per 100,000 women per year with one lifetime screening, and 6.01 per 100,000 women per year with vaccination), but more effective than no prevention (17.45 CC cases per 100,000 women per year). The most effective and expensive strategy is vaccination combined with three lifetime screenings for all women (2.74 CC cases per 100,000 women for a cost of $303,324 for a 3-dose vaccine and $242,523 for a 2-dose vaccine).

**Table 2:** Costs and clinical outcomes for women under each prevention strategy*

**Scenario investigated**	Annual cost for 100,000 women (per 1 Woman)^**^	**Annual CC cases for 100,000 women**
No prevention	$26,201 ($0.26)	17.45
One lifetime screening	$75,418 ($0.75)	12.15
Vaccination (3 doses)	$191,415 ($1.91)	6.01
Vaccination (3 doses) and one lifetime screening	$238,580 ($2.39)	4.25
Two lifetime screenings (sensitivity analysis only)	$115,082 ($1.15)	9.63
Three lifetime screenings (sensitivity analysis only)	$145,316 ($1.45)	7.85
Vaccination (3 doses) and two lifetime screenings (sensitivity analysis only)	$275,519 ($2.76)	3.39
Vaccination (3 doses) and three lifetime screenings (sensitivity analysis only)	$303,324 ($3.03)	2.74
Vaccination (2 doses)	$130,603 ($1.31)	6.01
Vaccination (2 doses) and one lifetime screening	$177,775 ($1.78)	4.25
Vaccination (2 doses) and two lifetime screenings (sensitivity analysis only)	$214,717 ($2.15)	3.39
Vaccination (2 doses) and three lifetime screenings (sensitivity analysis only)	$242,523 ($2.43)	2.74

*Inputs for the linear programming model.

**100% coverage, all women undergoing the specified strategy.

### Optimization model: base case

**Figure 2:**
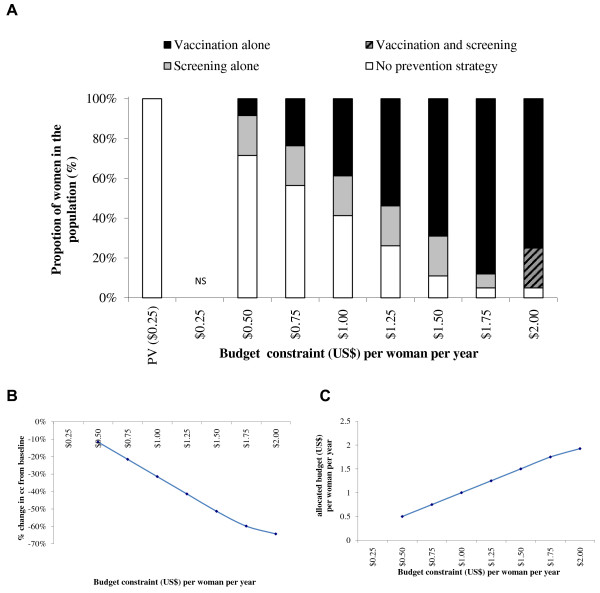
**Optimal mix of prevention strategies (A), associated CC reduction (B) and allocated budget/expenditure (C).** Upper-Bound Coverage of 20% screening and 95% vaccination (3-dose vaccination schedule). NS = No solution found; PV = Pre-vaccination schedule. Note: There is only a one lifetime screening option for screening.

**Figure 3:**
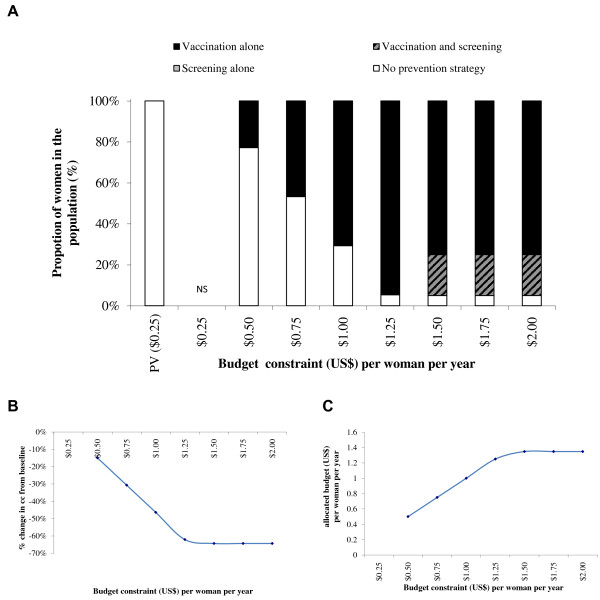
**Optimal mix of prevention strategies (A), associated CC reduction (B) and allocated budget/expenditure (C).** Upper-Bound Coverage of 20% screening and 95% vaccination (2-dose vaccination schedule). NS = No solution found; PV = Pre-vaccination schedule. Note: There is only a one lifetime screening option for screening.

Figure 2A presents the optimal allocation of resources for screening and vaccination in Nigeria at different budget constraints (i.e., the maximum levels of expenditure per woman per year for the prevention and treatment of CC) with a 20% coverage limit for one lifetime screening and a 95% coverage limit for vaccination with a 3-dose vaccination schedule. The stacked columns represent the estimated optimal proportion of women in the population receiving each intervention in order to reach the maximum CC reduction compared with pre-vaccination. Figure 2B presents the percentage reduction in CC cases from the pre-vaccination value of 17.45 per 100,000 women when the optimal allocation of resources to screening and vaccination is achieved at different levels of budget constraint. Figure 2C presents the budget associated with the optimal strategies for each set of constraints included in the optimization model under the base-case. At maximum constraint values of a budget of $1.00 per woman and coverage of 95% for vaccination and 20% for one lifetime screening, the optimal strategy would be 39% with vaccination alone, 20% with one lifetime screening, 0% with vaccination and one lifetime screening, and 41% with no prevention strategy. This would result in a 31% reduction in the number of CC cases. With a 2-dose vaccination schedule, as presented in Figure 3, the optimal mix of prevention strategies at the $1 per woman budget constraint would be 71% with vaccination alone, 0% with one lifetime screening, 0% with vaccination plus one lifetime screening, and 29% with no prevention strategy. This would result in a 46% reduction in the number of CC cases.

With a budget constraint of $2.00 per woman per year, the maximum prevention coverage could be reached and the optimal mix of prevention strategies to minimize CC incidence would be (with both a 2- and a 3-dose vaccination schedule), 75% with vaccination alone, 20% with vaccination and one lifetime screening, and 5% with no prevention strategy. This would result in a CC reduction of 64% with both a 3-dose and a 2-dose vaccination schedule. The resulting expenditure would be $1.93 (3-dose vaccination schedule) and $1.35 (2-dose vaccination schedule) per woman per year.

**Figure 4:**
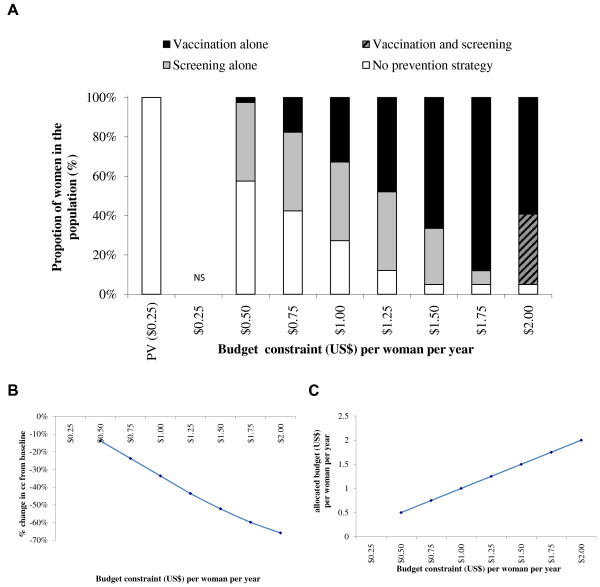
**Optimal mix of prevention strategies (A), associated CC reduction (B) and allocated budget/expenditure (C).** Upper-Bound Coverage of 40% screening and 95% vaccination (3-dose vaccination schedule). NS = No solution found; PV = Pre-vaccination schedule. Note: There is only a one lifetime screening option for screening.

**Figure 5:**
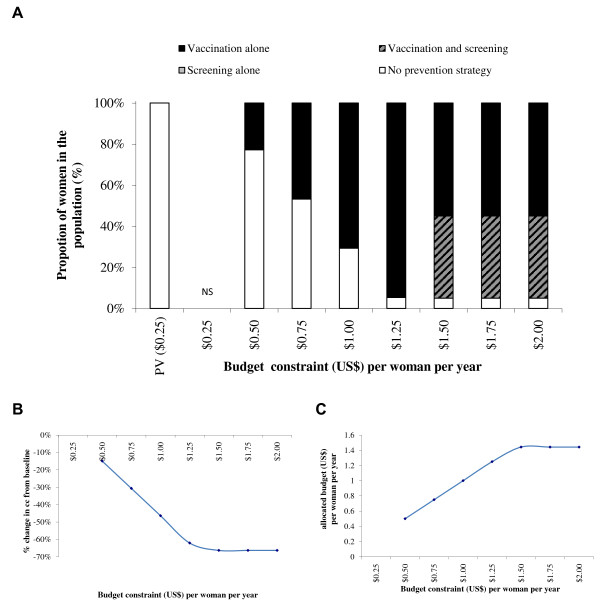
**Optimal mix of prevention strategies (A), associated CC reduction (B) and allocated budget/expenditure (C).** Upper-Bound Coverage of 40% screening and 95% vaccination (2-dose vaccination schedule). NS = No solution found; PV = Pre-vaccination schedule. Note: There is only a one lifetime screening option for screening.

**Figure 6:**
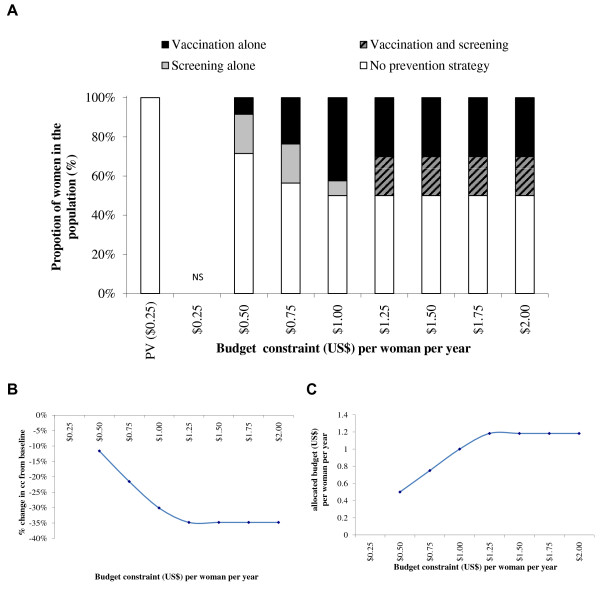
**Optimal mix of prevention strategies (A) associated CC reduction (B) and allocated budget/expenditure (C).** Upper-Bound Coverage of 20% screening and 50% vaccination (3-dose vaccination schedule). NS = No solution found; PV = Pre-vaccination schedule. Note: There is only a one lifetime screening option for screening.

**Figure 7:**
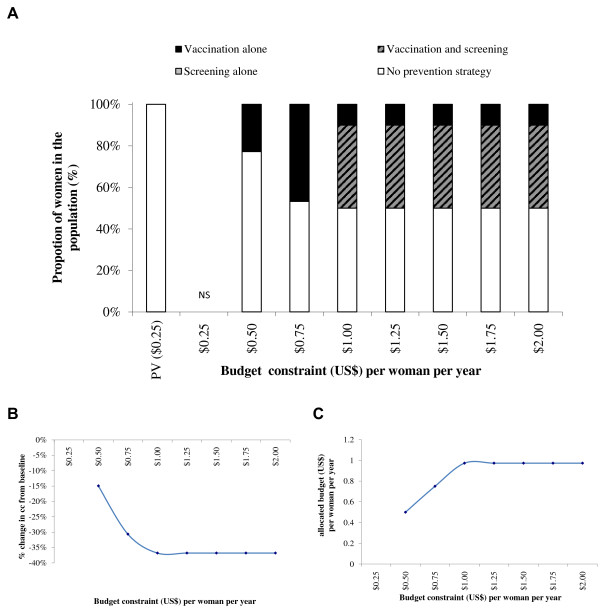
**Optimal mix of prevention strategies (A) associated CC reduction (B) and allocated budget/expenditure (C).** Upper-Bound Coverage of 20% screening and 50% vaccination (2-dose vaccination schedule). NS = No solution found; PV = Pre-vaccination schedule. Note: There is only a one lifetime screening option for screening.

Although the most effective of the four strategies included in the base case is vaccination plus one lifetime screening, the optimal mix of prevention strategies does not include this combination until the budget constraint per woman is set to $2.00 with a 3-dose vaccination schedule and $1.50 with a 2-dose vaccination schedule.Figures 4, 5, 6 and 7 show similar impacts of relaxing the budget constraint within different constraints on one lifetime screening and vaccination coverage. In Figures 4 and 5, representing a scenario with a vaccination coverage constraint of 95% and once in a lifetime screening coverage constraint of 40%, the maximum coverage of prevention strategies would result in an expenditure of $2.02 (3-dose vaccination schedule) to $1.44 (2-dose vaccination schedule) per woman, with an associated 66% reduction of incident CC cases. In Figures 6 and 7, which present a scenario with a vaccination coverage constraint of 50% and a screening coverage constraint of 20%, the maximum prevention coverage would result in an expenditure of $1.18 (3-dose vaccination schedule) to $0.88 (2-dose vaccination schedule) per woman per year, and an associated 35% reduction in CC cases.

With a 3-dose vaccination schedule (Figures 2, 4 and 6) the optimal mix of prevention strategies includes screening alone for the lowest budget constraint. With a 2-dose vaccination schedule (Figures 3, 5 and 7), the lowest budget constraints do not include screening but include vaccination alone. Screening is only part of the optimal mix of prevention strategies for budget constraints of at least $1.50 per woman per year with a vaccination coverage constraint of 95% or at least $1.00 per woman per year with a vaccination coverage constraint of 50%.

### Sensitivity analysis

One way sensitivity analyses were performed for a budget constraint of $1 and $2 per woman per year. The costs of screening and treating CIN grade 1, CIN grades 2 and 3, and CC were varied, as was the frequency of lifetime screenings and the duration and level of protection resulting from vaccination. An additional scenario investigated the use of a HPV test as the screening method instead of the Pap test. The results of the sensitivity analyses, measured as the percentage of CC cases prevented compared with the pre-vaccination incidence of CC cases (17.45 per 100,000 women) are shown in Table 3 for a budget constraint of $1 per woman per year and Table 4 for a budget constraint of $2 per woman per year. The results indicate that the maximum reachable CC reduction was higher with a 2-dose than with a 3-dose vaccination schedule under all sensitivity analyses conducted. The optimal mix of strategies under the different sensitivity analyses are presented in the Additional file 1: Figure S1 and Additional file 2: Figure S2. The costs of CIN and CC treatment, as well as the vaccine characteristics, had the largest impact on the optimal CC reduction. A low cost led to a higher coverage of the population by a prevention strategy resulting in more CC cases prevented, while a high cost led to a lower coverage and hence a lower CC reduction. Interestingly, the optimal strategy with a high cost for treating precancerous lesion would imply vaccination alone or no prevention with either a 3- or a 2-dose vaccination schedule. However, the optimal strategy with a high cost for treating cancer would combine screening alone, vaccination alone and no prevention with a 3-dose vaccination schedule, and vaccination alone, vaccination combined with screening or no prevention with a 2-dose vaccination schedule. The use a 2-dose vaccine with a reduction in the vaccine efficacy also led to a lower CC reduction under the optimal mix of strategies and a combination of screening and vaccination with however a larger vaccination coverage than with a 3-dose vaccination. The use of a HPV test for the screening assuming a lower costs and higher sensitivity led to a higher CC reduction while the optimal mix would combine both screening and vaccination.

**Table 3:** Annual CC cases and change from the pre-vaccination situation under the optimal budget allocation

**Sensitivity analyses scenario**	**Screening and vaccine coverage constraints**		
	**20% screening, 95% vaccination**	**40% screening, 95% vaccination**	**20% screening, 50% vaccination**
***Base case (3 doses)***			
Base case—mean treatment costs and one lifetime screening	12.0 (-31%)	11.6 (-34%)	12.1 (-31%)
Prevention cost minus 20% and CIN treatment costs minus 1 SD	11.6 (-34%)	10.8 (-38%)	12.3 (-29%)
Prevention costs plus 20% and CIN treatment costs plus 1 SD	12.3 (-29%)	12.3 (-29%)	12.1 (-31%)
CC treatment costs minus 1 SD	10.7 (-38%)	10.4 (-40%)	11.7 (-33%)
CC treatment costs plus 1 SD	13.5 (-23%)	13.1 (-25%)	13.5 (-23%)
Allow scenarios with two lifetime screenings	12.0 (-31%)	11.6 (-34%)	12.1 (-30%)
Allow scenarios with two or three lifetime screenings	12.0 (-31%)	11.6 (-34%)	12.1 (-31%)
Screening using HPV test	11.6 (-34%)	10.8 (-38%)	12.0 (-31%)
Vaccine (3 doses) duration of protection = 25 years, vaccine efficacy reduced by 20%	12.8 (-27%)	12.3 (-30%)	13.1 (-25%)
Alternative scenario (2 doses)^*^			
Base case mean treatment costs and one lifetime screening	9.4 (-46%)	9.4 (-46%)	11.0 (-37%)
Prevention cost minus 20% and CIN treatment costs minus 1 SD	8.8 (-50%)	8.4 (-52%)	11.0 (-37%)
Prevention costs plus 20% and CIN treatment costs plus 1 SD	9.4 (-46%)	9.4 (-46%)	11.1 (-36%)
CC treatment costs minus 1 SD	7.9 (-55%)	7.9 (-55%)	11.0 (-37%)
CC treatment costs plus 1 SD	11.4 (-35%)	11.4 (-35%)	11.6 (-33%)
Allow scenarios with two lifetime screenings	9.4 (-46%)	9.4 (-46%)	11.0 (-37%)
Allow scenarios with two or three lifetime screenings	9.4 (-46%)	9.4 (-46%)	11.0 (-37%)
Screening using HPV test	8.8 (-49%)	8.5 (-51%)	11.3 (-35%)
Vaccine (2 doses) duration of protection = 25 years, vaccine efficacy reduced by 20%	10.8 (-38%)	10.6 (-39%)	12.3 (-29%)

Sensitivity analyses: budget constraint $1 (~4 times pre-vaccination budget) per woman.

V-S(int)-V&S-None.

CIN = Cervical intraepithelial neoplasia; CC = Cervical cancer; SD = Standard deviation.

*assume, in the base case, the same efficacy for a three or a two dose vaccine.

**Table 4:** Annual CC cases and change from the pre-vaccination situation under the optimal budget allocation

**Sensitivity analyses scenarios**	**Screening and vaccine coverage constraints**		
	**20% screening, 95% vaccination**	**40% screening, 95% vaccination**	**20% screening, 50% vaccination**
***Base case (3 doses)***			
Base case—mean treatment costs and one lifetime screening	6.2 (-64%)	6.0 (-66%)	11.4 (-35%)
Prevention cost plus 20% and CIN treatment costs plus 1 SD	6.2 (-64%)	5.9 (-66%)	11.4 (-35%)
Prevention costs minus 20% and CIN treatment costs minus 1 SD	6.2 (-64%)	6.1 (-65%)	11.4 (-35%)
CC treatment costs plus 1 SD	6.2 (-64%)	5.9 (-66%)	11.4 (-35%)
CC treatment costs minus 1 SD	6.2 (-64%)	6.3 (-64%)	11.4 (-35%)
Allow scenarios with two lifetime screenings	6.1 (-65%)	6.0 (-66%)	11.2 (-36%)
Allow scenarios with two or three lifetime screenings	5.9 (-66%)	6.0 (-66%)	11.1 (-37%)
Screening using HPV test	6.2 (-65%)	5.8 (-67%)	11.3 (-35%)
Vaccine duration of protection = 25 years, vaccine efficacy reduced by 20%	7.3 (-58%)	7.1 (-59%)	12.0 (-31%)
Alternative scenario (2 doses)^*^			
Base case—mean treatment costs and one lifetime screening	6.2 (-64%)	5.9 (-66%)	11.4 (-35%)
Prevention cost plus 20% and CIN treatment costs plus 1 SD	6.2 (-64%)	5.9 (-66%)	11.4 (-35%)
Prevention costs minus 20% and CIN treatment costs minus 1 SD	6.2 (-64%)	5.9 (-66%)	11.4 (-35%)
CC treatment costs plus 1 SD	6.2 (-64%)	5.9 (-66%)	11.4 (-35%)
CC treatment costs minus 1 SD	6.2 (-64%)	5.9 (-66%)	11.4 (-35%)
Allow scenarios with two lifetime screenings	6.1 (-65%)	5.5 (-68%)	11.2 (-36%)
Allow scenarios with two or three lifetime screenings	5.9 (-66%)	5.3 (-70%)	11.1 (-37%)
Screening using HPV test	6.2 (-65%)	5.8 (-67%)	11.3 (-35%)
Vaccine duration of protection = 25 years, vaccine efficacy reduced by 20%	8.1 (-54%)	7.7 (-56%)	12.3 (-29%)

Sensitivity analyses: budget constraint $2 (~8 times pre-vaccination budget) per woman.

CIN = Cervical intraepithelial neoplasia; CC = Cervical cancer; SD = Standard deviation.

*assume, in the base case, the same efficacy for a three or a two dose vaccine.

With a $2 per woman budget constraint, the maximum vaccination and screening coverage is reached under the optimal mix of strategies for all sensitivity analyses performed, and hence the maximum CC reduction is reached with both the 3-dose and the 2-dose vaccination schedule scenarios and under all sensitivity analyses.

## Discussion

We have developed a model that would identify the optimal mix of CC prevention strategies (screening and or vaccination) to minimize the number of CC cases for different scenarios defined by constraints on budget, maximum screening and vaccination coverage, and overall reachable population. Under the base case, three scenarios were considered for either a 3-dose or a 2-dose potential vaccination schedule to capture multiple alternatives regarding the available budget and feasibility of implementation in Nigeria.

### Main findings

The results of the Markov evaluation models indicated that the number of CC cases expected from a 100% coverage of each prevention strategy was lowest for vaccination (6.01 per 100,000 women per year) compared with one lifetime screening (12.15 per 100,000 women per year), two lifetime screenings (9.63 per 100,000 women per year), three lifetime screenings (7.85 per 100,000 women per year), or no prevention (17.45 per 100,000 women per year).

In the base-case optimization model analyses, with upper-bound coverage constraints of 20% for screening and of 95% for vaccination and a budget constraint at $1 per woman, the optimal mix of prevention strategies would result in a 31% CC reduction compared with today’s CC incidence with a 3-dose vaccination schedule, and in a 46% CC reduction with a 2-dose vaccination schedule. With a 3-dose vaccination schedule, the optimal combination would be 20% with screening alone, 39% with vaccination alone and 41% without any prevention, while with a 2-dose vaccination schedule the optimal combination would be 0% screened, 71% vaccinated, and 29% without any prevention. Under the lower budget constraints, the optimal strategy with a 3-dose vaccination schedule would always be a combination of screening alone, vaccination alone and no prevention, while with a 2-dose vaccination schedule the optimal combination would only include vaccination and no prevention by screening. Using an increment in budget constraint of $0.25 per women per year going from $0.25 to $2.00, any budget constraint equal to or higher than $2.00 per woman with a 3-dose vaccination schedule, or $1.50 per woman with a 2-dose vaccination schedule, would result in the optimal strategy with a maximum CC reduction using a strategy consisting of 75% with vaccination alone, 20% with vaccination and screening and 5% without prevention. The associated CC reduction would be 64%. These strategies would be specifically associated with a budget of $1.93 per woman with a 3-dose vaccination schedule and $1.35 per woman with a 2-dose vaccination schedule.

The results were similar for different screening and vaccination coverages, except that in the case of an upper-bound vaccination coverage of 50%, the percentage reduction in CC cases compared with the pre-vaccination situation had a maximum value of 35% at an expenditure of $1.18 per woman with a 3-dose vaccination schedule and $0.88 per woman with a 2-dose vaccination schedule. Sensitivity analyses indicated that the results were sensitive to the CIN and CC treatment cost. Higher treatment costs resulted in lower achievable coverage with vaccination and screening within the $1 budget constraint, and hence a lower reduction in the number of CC cases. With a 2-dose vaccination schedule, all sensitivity analyses tended to first maximize the vaccination coverage. With a 3-dose vaccination schedule, most sensitivity analyses (except a high CIN treatment cost) tended to maximize both vaccination and screening and in all cases resulted in a lower reduction in CC than the reduction obtained with a 2-dose vaccination schedule.

The results from the optimization model indicate that when introducing a prevention program for CC, in a country like Nigeria where none is currently in place, with a 2-dose vaccination schedule it would be optimal to invest in vaccination alone to the extent feasible, followed by vaccination plus one lifetime screening. With a 3-dose vaccination schedule, a combination of both vaccination and screening would be optimal. This result depends therefore on the relative costs of vaccination and screening as well as the relative efficacy between the two interventions. The optimization would favor a strategy with a low costs and a high efficacy (sensitivity for the screening).

A similar model was recently used to estimate the optimal mix of CC screening and HPV vaccination in the UK and Brazil [30]. These two countries already had a screening program in place, with coverage levels of 65% in the UK and 50% in Brazil, each with a screening frequency of 3 years applied to all screened women. The optimization model indicated that keeping the existing screening program while extending the screening interval to 6 years and adding vaccination with 80% coverage would result in a reduction in CC cases of 41% in the UK and 54% in Brazil, with no change in expenditure per woman compared with the pre-vaccination situation. This is very different from the situation observed in Nigeria, where no nationwide screening programs are currently in place and one lifetime screening is standard for the few women who are screened. As a result, reducing CC in Sub-Saharan Africa will require that additional resources be spent per woman on either screening or HPV vaccination programs.

The current evaluation estimated that an optimal allocation of a CC prevention budget of $1 per woman per year could lead to an important reduction in CC burden by 31% to 46%, depending on the scenario chosen. In Nigeria, three tiers of Government (Federal, State and Local) have responsibility for health and decide about budget allocation. The current evaluation suggests that a combination of 2-dose vaccination with or without one lifetime screening is expected to lead to an important reduction in CC.

Nationwide deployment of HPV vaccines to reduce the CC burden in Africa is more realistic than implementing a screening program, given the current failure of the national screening program due to logistic and funding challenges. Most African countries have efficient and very well accepted national childhood immunization programs, as in Nigeria. HPV vaccination could be integrated into these programs, provided some operational challenges linked to the adaptation of the program to a different target population (adolescent for the HPV vaccination) are addressed. The cost of implementation would also be drastically reduced compared to that expected from the implementation of a screening program. However, in each country the age at which the vaccination is implemented should be adapted based on their national average age of girls’ sexual debut.

### Limitations of the optimization analysis

The optimization model used for this analysis has its limitations. First, the model outcome is obtained when an optimal mix of different prevention strategies is applied in the population and a steady-state incidence of CC is achieved across the population. Many years will elapse before reaching that state. The results therefore represent an optimal “ideal” situation that provides a direction to be followed but not a result that will occur immediately, and nor does it address specific implementation issues. Vaccination will affect the girls who will become the women of the future. Today’s women would not benefit from vaccination, but could directly benefit from a single lifetime screening. The vaccinated girls would not need any screening in the years post vaccination. This cost saving could be used to implement a screening program benefiting older women. This would accelerate the overall CC reduction. No discounting was applied in this analysis, as the model presents a steady-state evaluation over a 1-year period. Given the time lag needed to reach steady state, discounting could be considered, but the means of implementation and especially the value of including a discount rate in such an optimization exercise is debatable. A discounting applied to the input of the model down to a today value would not impact the optimization results while ways to implement the optimization on a population overtime still need to be investigated.

Second, a static Markov model was used to evaluate the cost and outcomes of each potential strategy at steady state across a population over a 1-year period. As the optimization model takes results from the evaluation model, the robustness of the optimization results therefore also depends upon the validity of the evaluation model. The Markov model used for this analysis did not account for dynamic effects of vaccination on infection, such as herd protection. It also assumed stability in the population demographic structure over time. However, using a dynamic model as the evaluation model instead of the static model would be more difficult to adapt to countries with limited data. It would also lead to nonlinearity between coverage and the outcomes, and thus would require the use of a nonlinear programming optimization model.

Third, the models presented here are deterministic: costs and effects in all combinations are known. It would be possible to characterize the allocation problem under conditions of uncertainty by reformulating the model as a stochastic analysis. However, existing approaches for such stochastic analyses are limited [49]. This is an area for future research, although the principal outcome of evaluating uncertainty is to indicate the need and the potential value of searching for more detailed and specific information [49].

Fourth, the results depend on the strategies initially investigated using the evaluation model. Therefore, including a vaccine with a different profile or a screening program with different characteristics (e.g., HPV testing instead of cytology-based screening) may lead to other optimal scenarios, as presented in the sensitivity analysis conducted in the current evaluation. Furthermore, the analysis does not take into account the possibility that the introduction of vaccination may affect the sensitivity of the screening test [50,51]. It also does not account for the cost associated with the implementation of a screening or vaccination program in Nigeria. Finally, our model assumes that the interventions evaluated are completely divisible in terms of intervals between screening tests and coverage. It ignores possible fixed set-up and implementation costs associated with interventions, which could constrain their divisibility. The fixed costs could be large for screening coverage, such as setting up the necessary infrastructure to collect and analyze samples, or implementing health education programs to increase awareness and overcome psychological and cultural barriers to uptake. Conversely, they may be relatively low for vaccine implementation in situations where other vaccination programs in the relevant age group are already established. In addition, the incremental implementation cost by coverage is unlikely to be linear. Disregarding such indivisible or nonlinear costs could be important in practice. Given the importance of the relative costs of vaccination and screening to the selection of the optimal program, the inclusion of these costs could have a large impact on the results.

## Conclusions

The optimization model presented here provides information needed to inform health policy decisions on the optimal allocation of limited resources to prevent CC most effectively. The results indicate that increased spending will be needed in Nigeria and in other countries in Sub-Saharan Africa that currently lack a CC prevention program, in order to achieve adequate prevention of CC cases. With a 3-dose vaccination schedule, the most efficient allocation of a limited budget would be to invest in both vaccination and screening, while with a 2-dose vaccination schedule investment should first be directed to vaccination before implementing a screening program.

## Note

All costs are in USD ($ in the text).

## Abbreviations

CC: Cervical cancer; CIN: Cervical intraepithelial neoplasia; CIN1: Cervical intraepithelial neoplasia, grade 1; CIN2/3: Cervical intraepithelial neoplasia, grades 2 and 3; HPV: Human papillomavirus; NGN: Nigerian naira; NS: No solution found; PAHO: Pan American Health Organization; Pap: Papanicolaou; PV: Pre-vaccination schedule; SD: Standard deviation; TNM: Classification of Malignant Tumours; UK: United Kingdom; VE: Vaccine efficacy; WHO: World Health Organization; 2LT: Two lifetime screenings; 3LT: Three lifetime screenings.

## Competing interests

IFA received financial support for conference attendance and speaking engagements from the GlaxoSmithKline group of companies and is serving on an Independent Data Monitoring Committee for an International HPV study.

ND is an employee of the GlaxoSmithKline group of companies and owns restricted shares in the GlaxoSmithKline group of companies.

IOMB and BA declare no conflict of interest.

## Authors’ contributions

ND conceived and designed the study, developed the model, conducted the analyses and was involved in the review and discussion of the results. IOMB, BA and IFA collected the data and were involved in the scheme analysis, review and discussion of the results. All authors reviewed and commented on drafts, and approved the final manuscript.

## Pre-publication history

The pre-publication history for this paper can be accessed here:

http://www.biomedcentral.com/1471-2407/14/365/prepub

## Supplementary Material

Additional file 1 **Optimal mix of strategies under the optimal budget allocation.** (a) vaccination overage 95%, screening coverage 20%; (b) vaccination coverage 95% screening coverage 40%; (c) vaccination coverage 50% screening coverage 20%. Sensitivity Analyses: Budget Constraint $1 (~4 times Pre-vaccination Budget) per Woman. CC = Cervical cancer; 2LT = Two lifetime screenings; 3LT = Three lifetime screenings; HPV = Human papillomavirus; VE = Vaccine efficacy. Click here for file

Additional file 2 **Optimal mix of strategies under the optimal budget allocation.** (a) vaccination overage 95%, screening coverage 20%; (b) vaccination coverage 95% screening coverage 40%; (c) vaccination coverage 50% screening coverage 20%. Sensitivity Analyses: Budget Constraint $2 (~8 times Pre-vaccination Budget) per Woman. CC = Cervical cancer; 2LT = Two lifetime screenings; 3LT = Three lifetime screenings; HPV = Human papillomavirus; VE = Vaccine efficacy. Click here for file
