# Supplementary figures and images for: Modeling optimal cervical cancer prevention strategies in Nigeria
Source: BMC Cancer. 2014 May 24;14:365. doi: 10.1186/1471-2407-14-365 (PMC4057561; doi:10.1186/1471-2407-14-365)

| 3-dose vaccine | 2-dose vaccine |
| --- | --- |
| (a)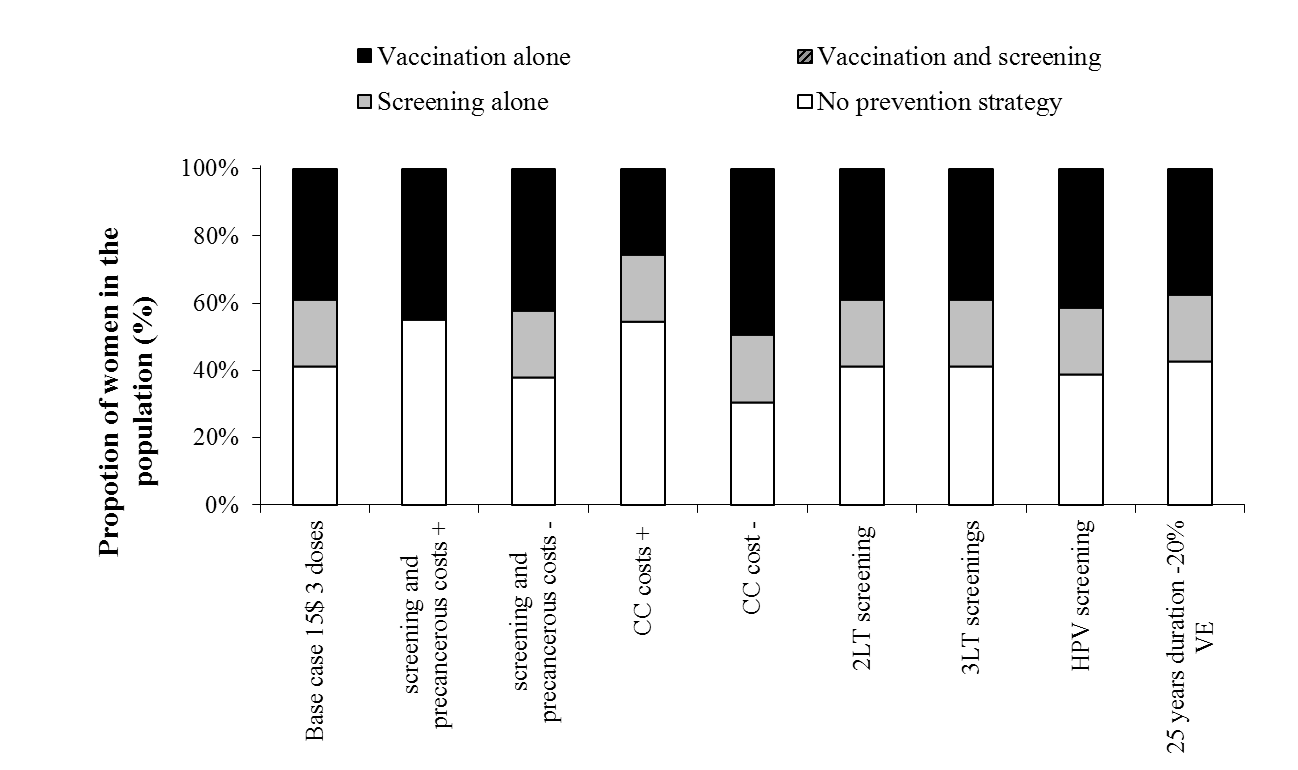 | (a)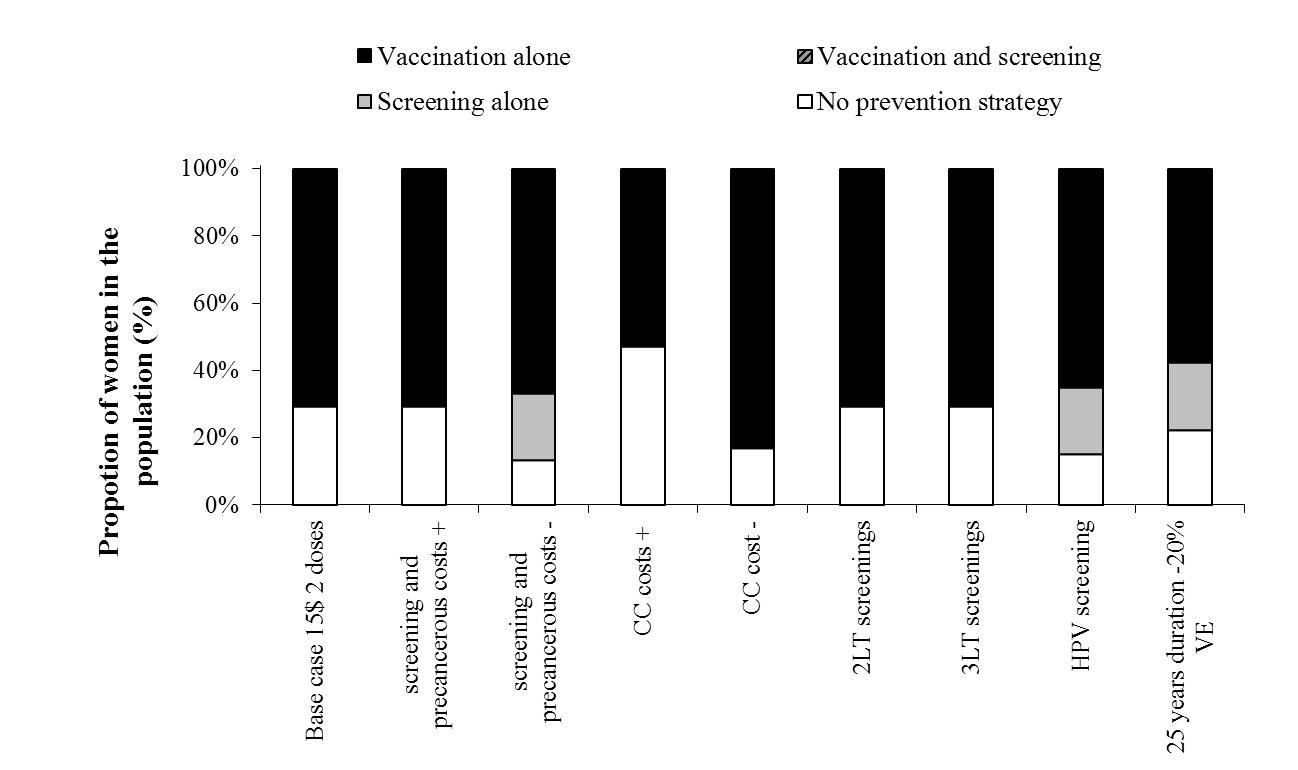 |
| (b)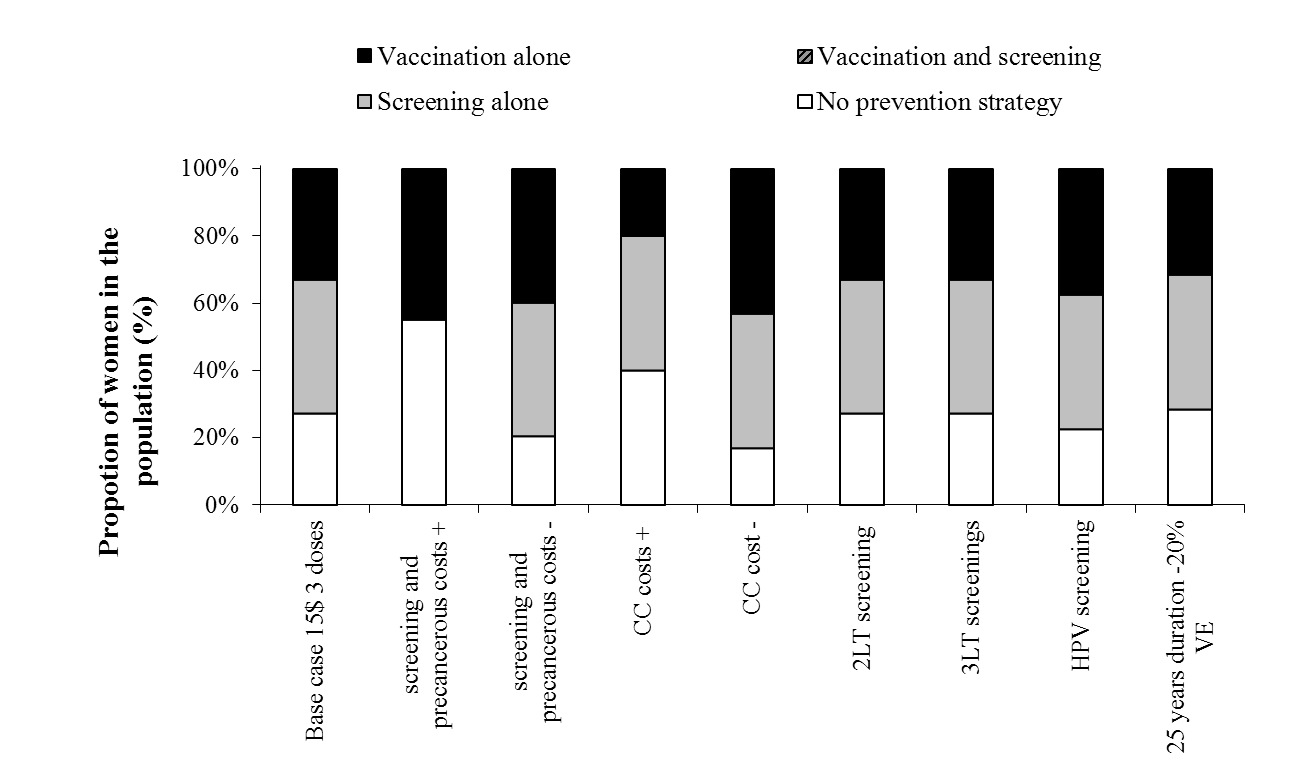 | (b)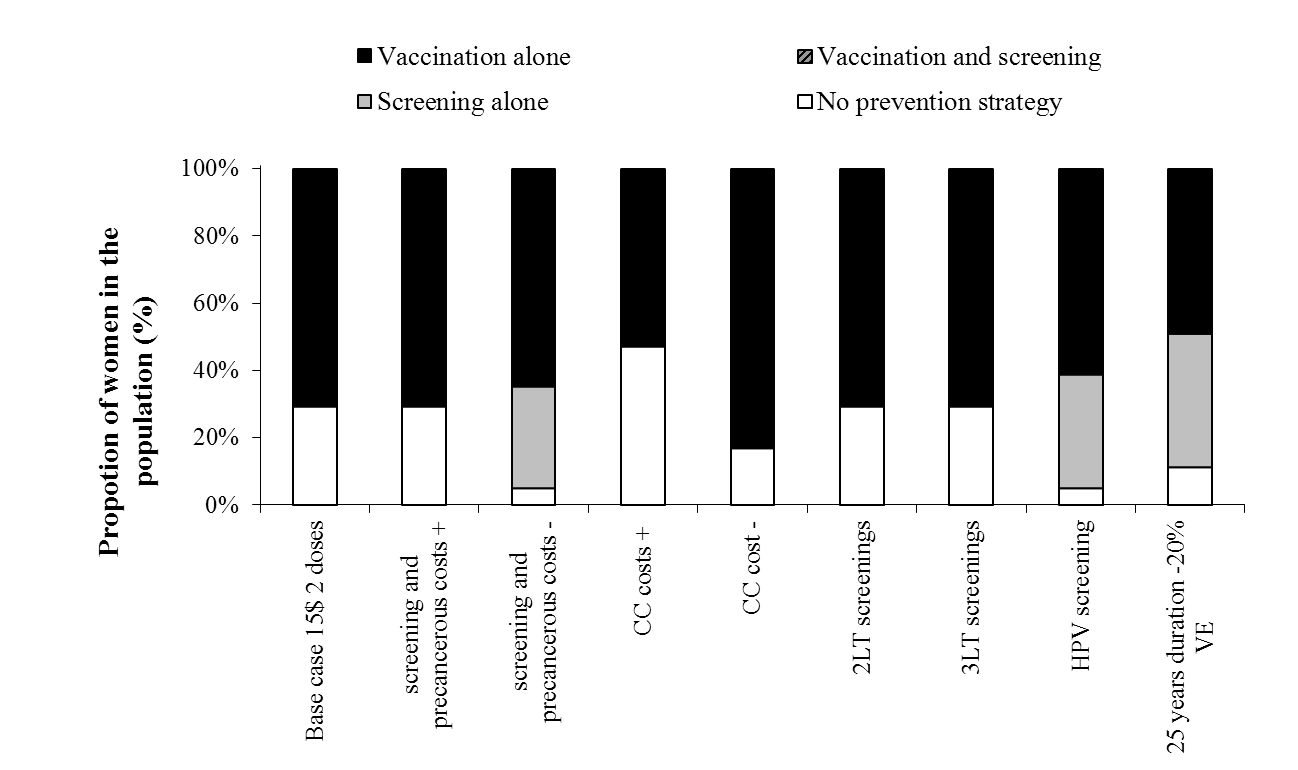 |
| (c)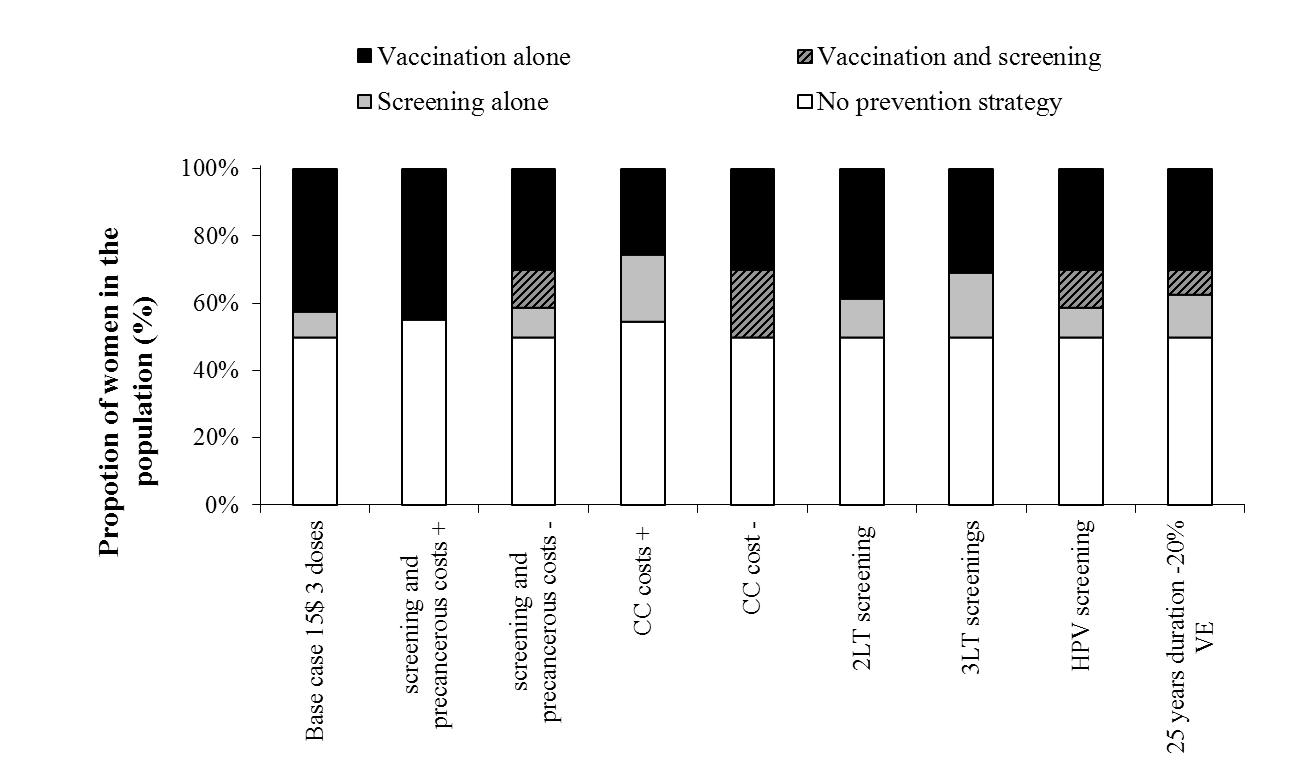 | (c)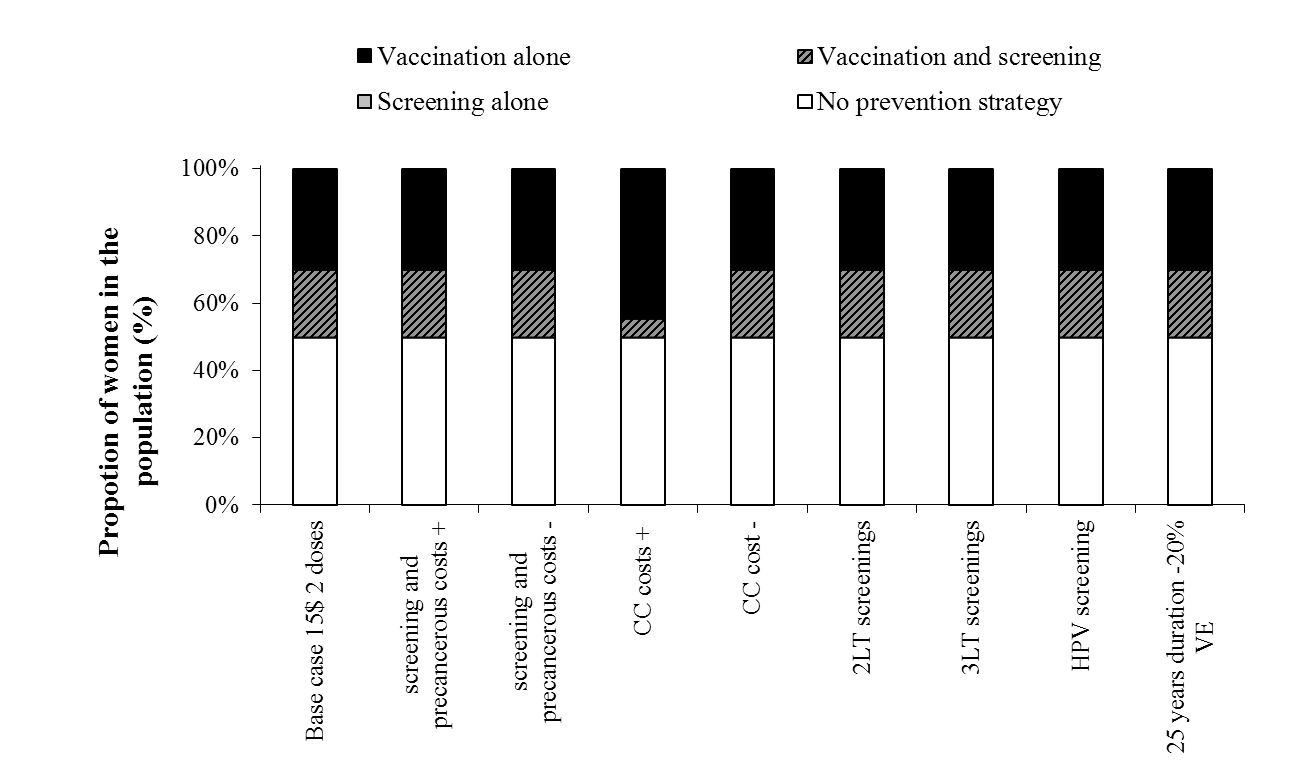 |

Supplement: Additional file 1 — Optimal mix of strategies under the optimal budget allocation. (a) vaccination overage 95%, screening coverage 20%; (b) vaccination coverage 95% screening coverage 40%; (c) vaccination coverage 50% screening coverage 20%. Sensitivity Analyses: Budget Constraint $1 (~4 times Pre-vaccination Budget) per Woman. CC = Cervical cancer; 2LT = Two lifetime screenings; 3LT = Three lifetime screenings; HPV = Human papillomavirus; VE = Vaccine efficacy. [file 1471-2407-14-365-S1.docx]

| 3-dose vaccine | 2-dose vaccine |
| --- | --- |
| (a) 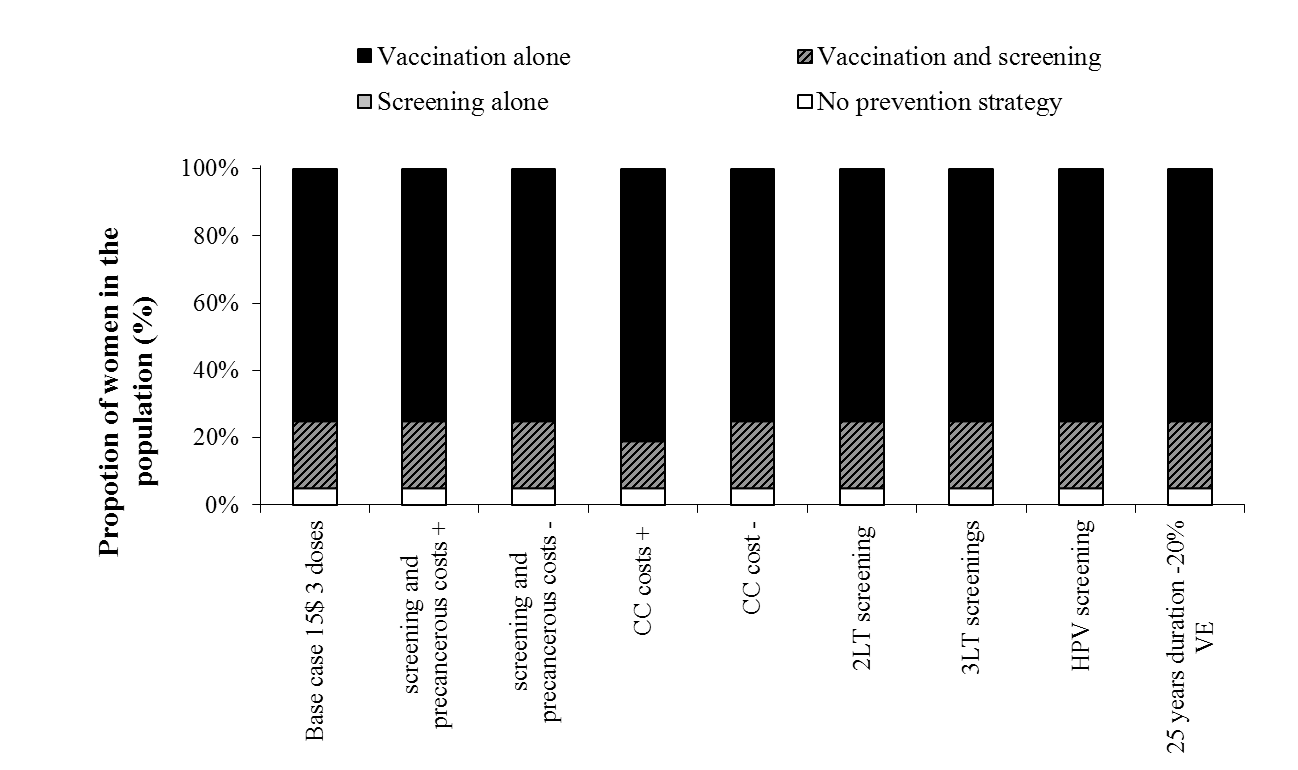 | (a)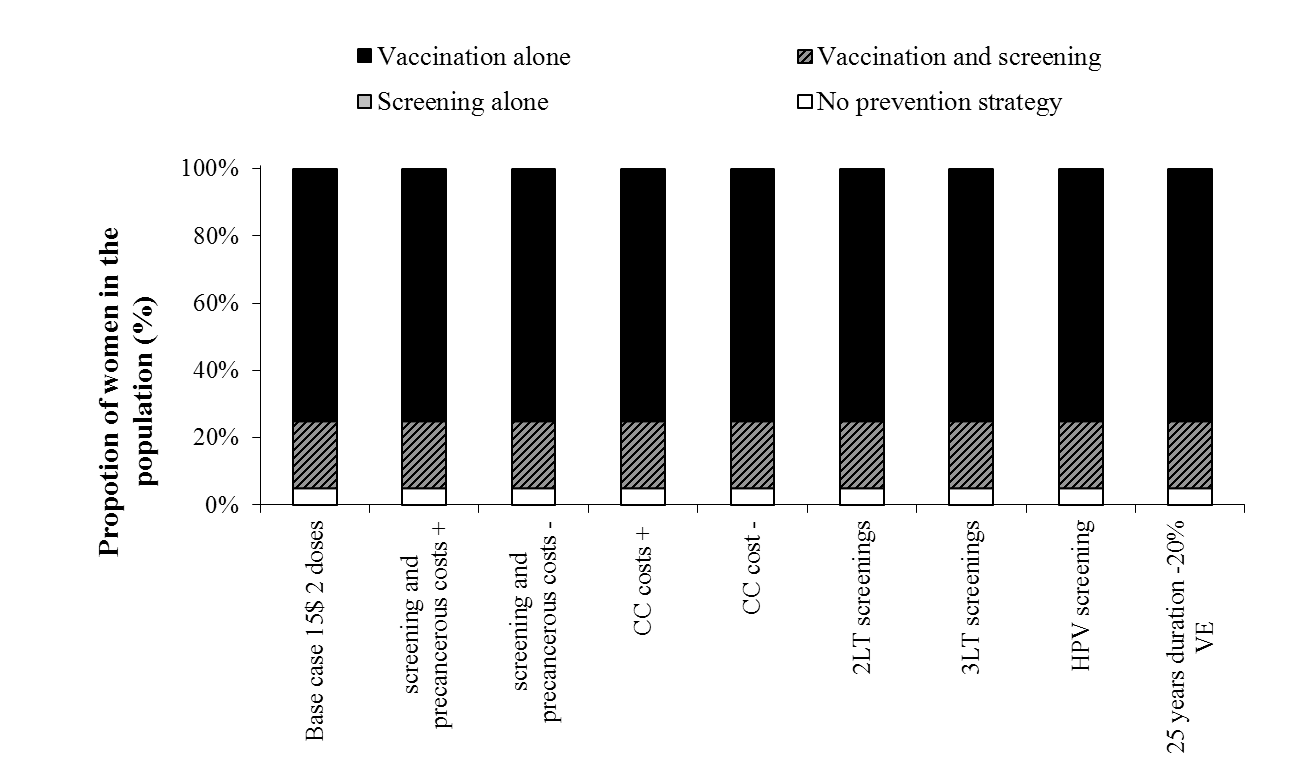 |
| (b)  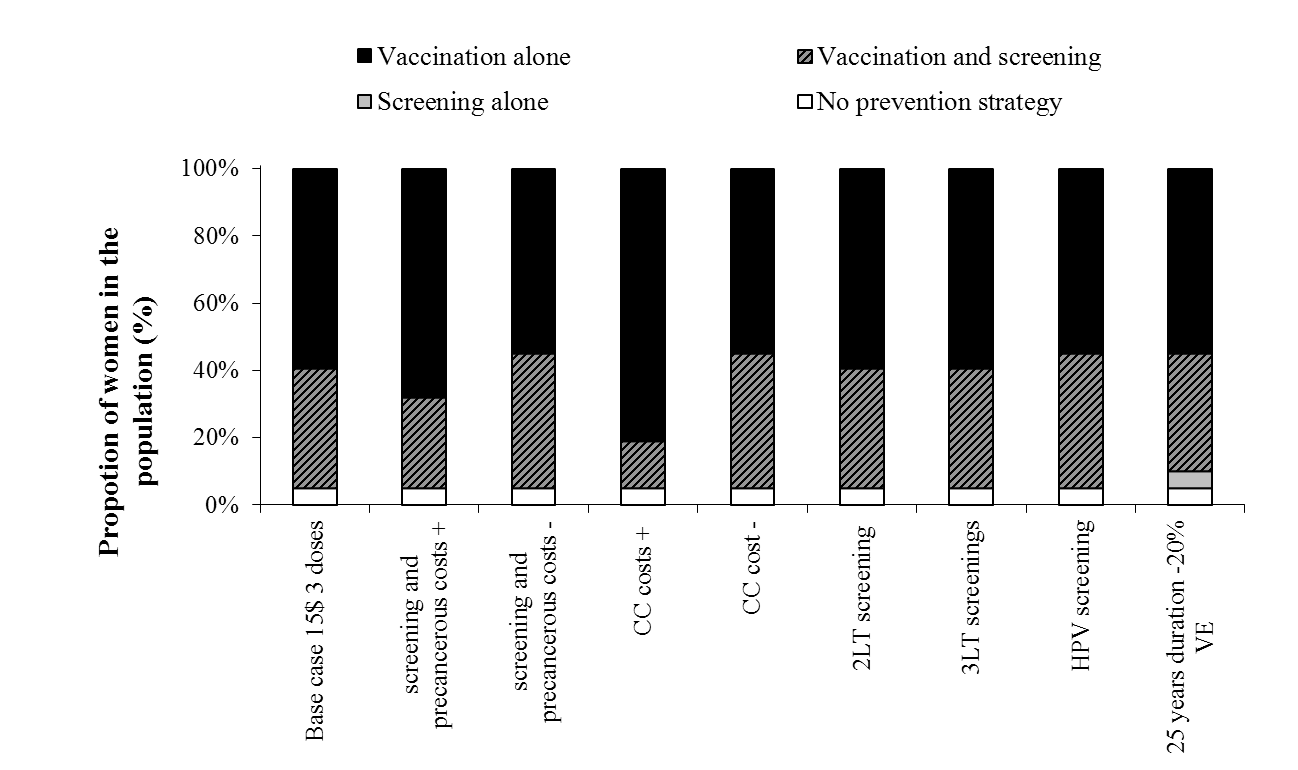 | (b)  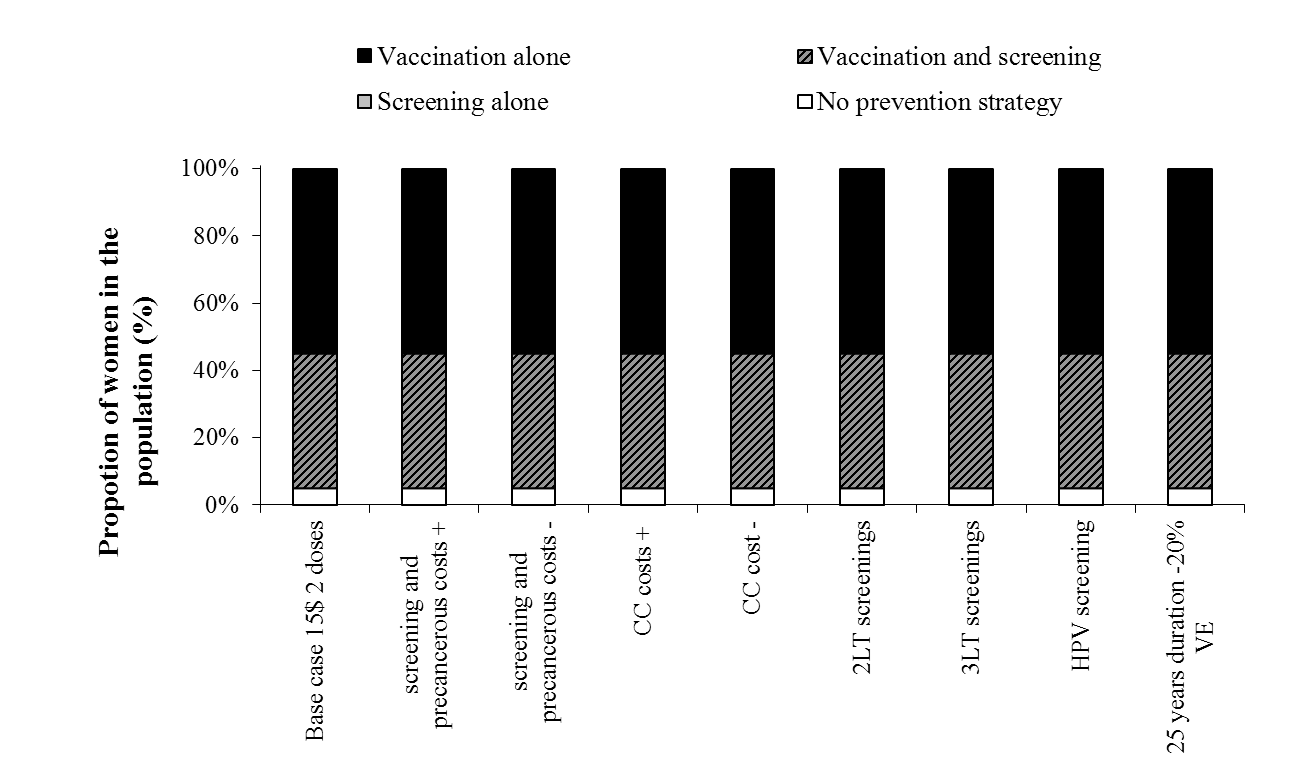 |
| (c)  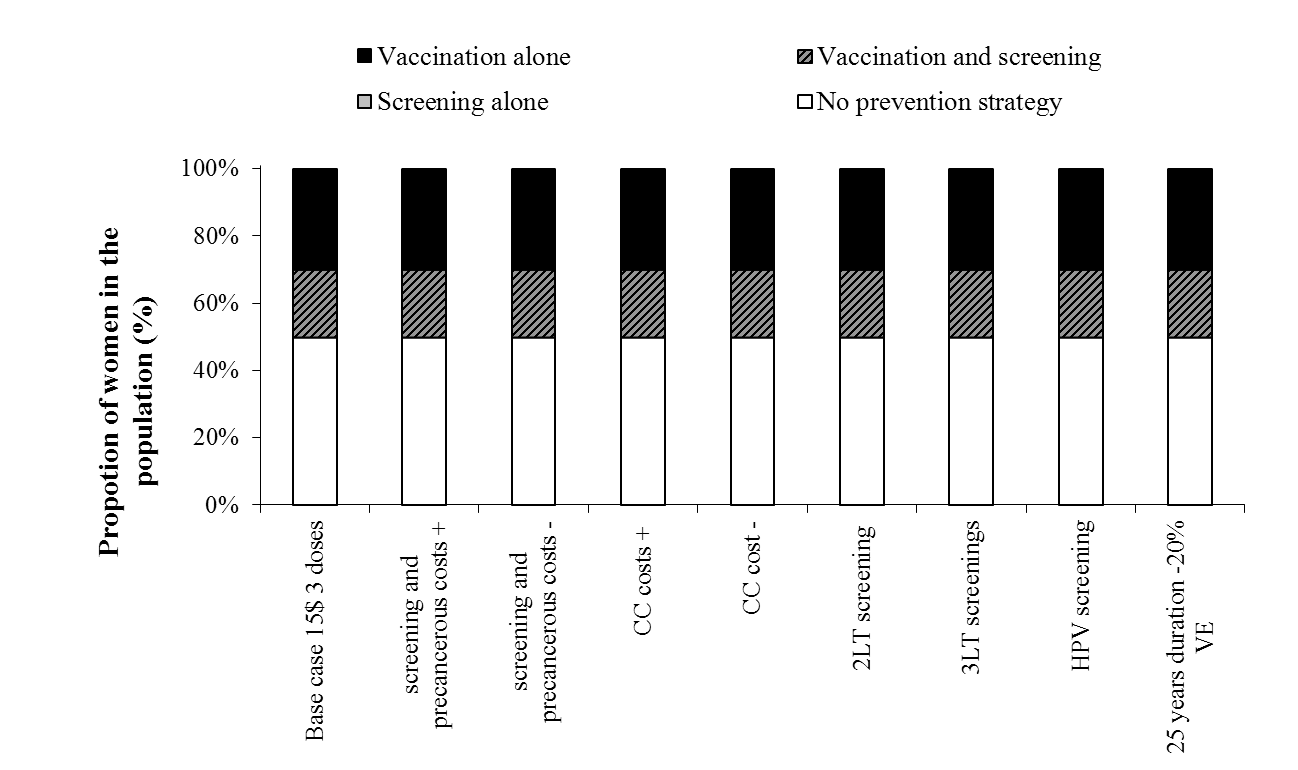 | (c)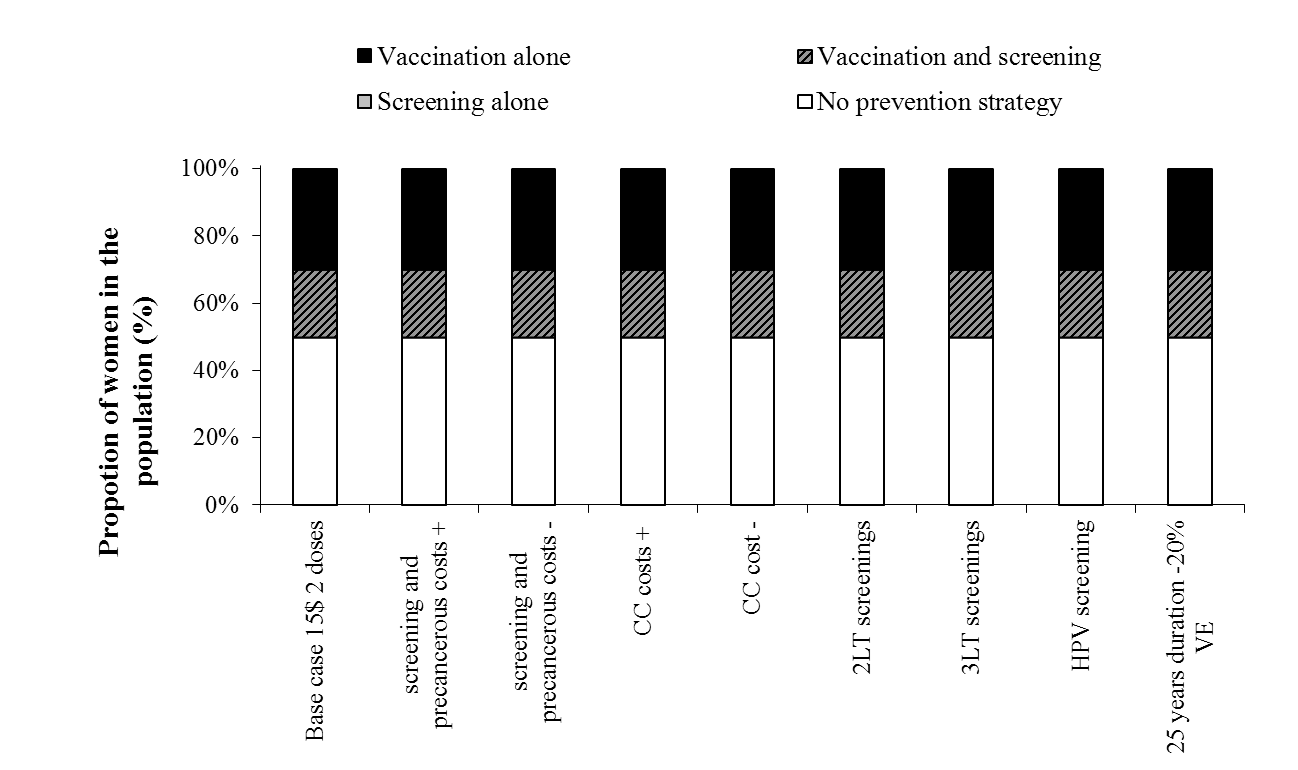 |

Supplement: Additional file 2 — Optimal mix of strategies under the optimal budget allocation. (a) vaccination overage 95%, screening coverage 20%; (b) vaccination coverage 95% screening coverage 40%; (c) vaccination coverage 50% screening coverage 20%. Sensitivity Analyses: Budget Constraint $2 (~8 times Pre-vaccination Budget) per Woman. CC = Cervical cancer; 2LT = Two lifetime screenings; 3LT = Three lifetime screenings; HPV = Human papillomavirus; VE = Vaccine efficacy. [file 1471-2407-14-365-S2.docx]
